# Oral-fluid inflammatory and oxidative-stress biomarkers for peri-implantitis in Asian adults: a systematic review and stratified random-effects meta-analysis

**DOI:** 10.1515/biol-2025-1360

**Published:** 2026-10-16

**Authors:** Lina Feng, Lei Zhang, Wenfeng He, Yuan Yuan, Hongxia Gao, Yanjun Ren, Xin Wang

**Affiliations:** Department of Stomatology, The Third Medical Centre of PLA General Hospital, No. 69, Yongding Road, Haidian District, Beijing 100039, Beijing, China

**Keywords:** peri-implantitis, peri-implant crevicular fluid, saliva, biomarkers

## Abstract

Peri-implantitis (PI) is an inflammatory condition where oral-fluid biomarkers can reflect disease – associated host responses. This meta – analysis evaluated biomarker differences between PI and healthy conditions in Asian adults, focusing on inflammatory cytokines (IL – 1β, IL – 6, TNF – α) and SIRT1 in peri – implant gingival crevicular fluid and saliva. Five databases were searched until August 2025. Random – effects models were used for pooled analyses, and relevant indicators were calculated. Thirteen studies were included. IL – 1β, IL – 6, and TNF – α were significantly higher in PI with low heterogeneity and stable findings. SIRT1 showed potentially favorable discriminatory performance but with limited evidence. Biomarker type affected effect – size variation, and mild small – study asymmetry for IL – 6 did not change overall conclusions. Overall, IL – 1β, IL – 6, and TNF – α can be candidate adjunctive biomarkers for PI assessment in Asian populations. SIRT1 is promising, but its diagnostic relevance conclusions are preliminary and need validation. Current evidence is limited. Further prospective studies in diverse populations and validation of clinically applicable thresholds and cost – effectiveness are needed before broader clinical translation.

## 1 Introduction

Peri-implantitis (PI) is a microbial biofilm-induced inflammatory condition characterized by soft-tissue inflammation and progressive peri-implant bone loss that can ultimately compromise implant survival and function [1], 2]. Although the current diagnostic standard for peri-implantitis, as defined by the 2017 World Workshop on the Classification of Periodontal and Peri-Implant Diseases and Conditions, relies on a combination of clinical indices – bleeding on probing (BOP), suppuration, increased probing depth (PD), and radiographic evidence of peri-implant bone loss – these measures primarily detect disease after irreversible tissue damage has occurred. Importantly, they are not effective for identifying early or subclinical peri-implant inflammation, highlighting the need for adjunctive biomarkers to improve early diagnosis and monitoring [3], 4]. The lag between biological onset and radiographic change, together with case heterogeneity (e.g., smoking, glycemic control, prosthetic design, maintenance), underscores the need for biological markers that capture active host–microbe interactions and may assist risk stratification, early detection, and treatment monitoring [5], 6].

Oral-fluid biomarkers – particularly peri-implant crevicular fluid (PICF) and saliva – offer a minimally invasive window into local and systemic inflammatory milieus. A substantial body of work has profiled inflammatory cytokines (e.g., interleukin-1β, interleukin-6, tumor necrosis factor-α) and matrix enzymes (e.g., MMP-8) in PICF, consistently demonstrating higher concentrations in PI compared with peri-implant health and, in some cases, peri-implant mucositis [5], 7]. Yet, prior reviews often pooled heterogeneous matrices, mixed periodontal and peri-implant conditions, or did not provide prediction intervals that convey the dispersion of true effects expected in future settings [8], 9]. In addition, evidence from Asian populations – where oral-health systems, lifestyles, and comorbidity patterns may differ – has not been synthesized with the same granularity as global analyses, especially when Chinese-language databases are omitted.

Recent systematic reviews and biomarker-focused studies have further suggested that peri-implant disease is not reflected only by classical inflammatory cytokines, but also by matrix-remodeling mediators, oxidative-stress pathways, and broader host-response signatures. This is important because peri-implantitis is biologically heterogeneous, and reliance on a single analyte may not fully capture disease activity. In addition, region-focused synthesis may be methodologically meaningful, as differences in smoking prevalence, glycemic burden, maintenance access, implant practices, and reporting patterns across Asian populations may influence both biomarker distributions and the apparent consistency of clinical contrasts [10], [11], [12].

Beyond classical inflammatory readouts, oxidative-stress and immune-regulatory pathways have attracted attention as potential modulators of peri-implant host response. Among these, Sirtuin-1 (SIRT1), a NAD^+^-dependent deacetylase, is mechanistically linked to repression of NF-κB–driven transcription, restraint of NLRP3 inflammasome activation, and support of osteogenic differentiation – functions that collectively argue for a homeostatic role at the bone–implant interface [10].

Methodological issues also impede translation. Studies vary in specimen type (PICF vs saliva), analysis unit (implant vs patient), diagnostic criteria (2017 World Workshop consensus vs earlier definitions), and assay platforms (ELISA vs multiplex) [11], 12]. These differences can inflate between-study heterogeneity or obscure clinically relevant signals. Robust synthesis therefore requires (i) harmonized effect directions across markers, (ii) random-effects models with prediction intervals, (iii) stratified analyses that separate PICF from saliva and patient- from implant-level data, and (iv) meta-regression to interrogate *a priori* moderators such as biomarker class, assay method, and diagnostic criteria.

Against this background, we conducted a preferred reporting items for systematic reviews and meta-analyses (PRISMA)-guided systematic review and stratified random-effects meta-analysis focusing on oral-fluid biomarkers in Asian adults with PI. The primary objective was to clarify how biomarker levels change in Asian PI patients compared with healthy controls.

Quantified changes in inflammatory cytokines – interleukin-1 beta (IL-1β), interleukin-6 (IL-6), and tumor necrosis factor alpha (TNF-α) – in peri-implant crevicular fluid (PICF) and saliva. Evaluated oxidative-stress/immune-regulatory markers, with emphasis on SIRT1 and synthesized its diagnostic performance using pooled area under the curve (AUC) estimates. Conducted subgroup analyses stratified by specimen type (PICF vs saliva), analysis unit (patient vs implant), and diagnostic criteria (2017 consensus vs non-consensus). Applied meta-regression to identify study-level moderators (biomarker class, assay method, sample size, publication year) influencing effect sizes. Examined heterogeneity, prediction intervals, and publication bias to determine the robustness and generalizability of results. Through these analyses, we aimed to provide a comprehensive evaluation of oral-fluid biomarker alterations in Asian PI patients and to assess their potential discriminatory relevance and adjunctive clinical applicability.

## 2 Methods

### 2.1 Literature search strategy

This systematic review and meta-analysis was conducted in accordance with the preferred reporting items for PRISMA 2020 guidelines [13]. The review protocol was not prospectively registered before study initiation. During the revision process, supplementary protocol registration was initiated (INPLASY202650139), and the registration information will be provided once available.

A comprehensive electronic search was performed across five databases: PubMed, Embase, Web of Science (WoS), Cochrane Library, and China National Knowledge Infrastructure (CNKI), from their inception to August 2025, without language restrictions. The search terms combined concepts of specimen type (peri-implant crevicular fluid, saliva), condition (peri-implantitis), and biomarkers. The search strategy combined terms related to specimen type (peri-implant crevicular fluid and saliva), disease condition (peri-implantitis), and biomarkers.

The detailed search strategies were as follows: PubMed, (“peri-implant crevicular fluid”[Title/Abstract] OR PICF[Title/Abstract] OR saliva[Title/Abstract]) AND (peri-implantitis[Title/Abstract]); Embase, (‘peri-implant crevicular fluid’:ab,ti OR PICF:ab,ti OR saliva:ab,ti) AND (‘peri-implantitis’:ab,ti); Web of Science, TS=(“peri-implant crevicular fluid” OR PICF OR saliva) AND TS=(peri-implantitis); Cochrane Library, (“peri-implant crevicular fluid” OR PICF OR saliva) AND (peri-implantitis); and CNKI, (篇关摘 = “种植体龈沟液” OR 篇关摘 = “PICF” OR 篇关摘 = “唾液”) AND (篇关摘 = “种植体周围炎”). In addition, the reference lists of all included studies were manually screened to identify any further eligible articles.

### 2.2 Inclusion and exclusion criteria (PICOS)

Studies were selected according to the PICOS framework:

Population (P): Asian Adults with peri-implantitis, peri-implant mucositis, or healthy implants.

Intervention/Exposure (I): Measurement of biomarkers (inflammatory cytokines or oxidative stress regulators) in peri-implant crevicular fluid (PICF) or saliva.

Comparator (C): Healthy peri-implant controls or patients without peri-implantitis.

Outcomes (O): Quantitative biomarker concentrations (mean ± SD) and/or diagnostic performance indices (ROC, AUC, sensitivity, specificity).

Study design (S): Observational clinical studies (cross-sectional, case-control, cohort).

Exclusion criteria were: (1) *in vitro* or animal studies; (2) reviews, case reports, conference abstracts; (3) lack of PICF/saliva data; (4) absence of extractable biomarker values or diagnostic performance outcomes.

### 2.3 Data extraction and management

Two reviewers independently screened all records (titles/abstracts, then full texts) and extracted data into a predesigned template. Extracted items included: study ID (author, year, country), population characteristics (sample size, age, sex), diagnostic criteria for peri-implantitis, specimen type (PICF or saliva), assay method (e.g., ELISA, multiplex), biomarkers measured, mean and standard deviation of biomarker levels, diagnostic cut-off values, AUC, sensitivity, specificity, and analysis unit (patient/implant). Disagreements were resolved by consensus with a third reviewer.

### 2.4 Risk of bias assessment

The methodological quality of included studies was assessed using the Joanna Briggs Institute (JBI) critical appraisal checklist for prevalence studies and the Newcastle–Ottawa Scale (NOS) for case-control studies [14], 15]. Studies were classified as low risk (≥6 points), moderate risk (4–5 points), or high risk (≤3 points). Risk-of-bias summary plots were generated using the robvis package in R.

### 2.5 Statistical analysis

All statistical analyses were performed in R (version 4.3.2) with the metafor and meta packages.

For continuous outcomes (biomarker concentrations), standardized mean differences (SMDs; Hedges’ g) with 95 % confidence intervals were calculated directly from reported group means, standard deviations, and sample sizes. No log transformation was applied to cytokine concentration data before SMD pooling. For diagnostic accuracy outcomes (SIRT1), reported AUC values were pooled on the logit scale using a random-effects model. Before pooling, the extracted cytokine dataset was checked at the row level against the prespecified fields for study label, comparison group, sample size, mean, standard deviation, biomarker unit, and effect direction. The SMDs were therefore calculated on the original reported concentration scale from mean, standard deviation, and sample-size data, without log transformation or unit-standardizing transformation before SMD estimation; the only logit transformation in the analysis was restricted to AUC pooling for SIRT1 diagnostic accuracy.

Subgroup analyses were performed by specimen type (PICF vs saliva), analysis unit (patient vs implant), and diagnostic criteria (2017 consensus vs non-consensus).

Meta-regression explored the influence of covariates including biomarker class (inflammatory vs oxidative-stress regulators), assay method, analysis unit, total sample size, and publication year.

To further examine robustness, influence diagnostics (Baujat plots and study influence statistics) were used to identify studies contributing disproportionately to heterogeneity or pooled effects. Sensitivity analyses were conducted through leave-one-out procedures, sequentially omitting each study to test the stability of summary estimates. Additional sensitivity analyses were also conducted by excluding small studies, defined as studies with a total sample size below 40 for the relevant cytokine comparison, and by excluding the study with the largest absolute SMD for each biomarker to assess whether a high-effect study drove the pooled estimate. The prespecified summary of these additional exclusion analyses is provided in Supplementary Table S1, with a graphical overview in Supplementary Figure S1.

Publication bias was evaluated by funnel plots and Egger’s regression test; when *k* ≥ 10, additional tests (trim-and-fill, PET-PEESE, and selection models) were applied.

A two-tailed *p*-value < 0.05 was considered statistically significant.

## 3 Results

### 3.1 Study selection and characteristics

A total of 1,282 records were retrieved from five databases, including PubMed (*n* = 276), Web of Science (*n* = 240), CNKI (*n* = 431), Embase (*n* = 320), and the Cochrane Library (*n* = 15). After removal of duplicates (*n* = 310), 972 unique records remained for title and abstract screening. Of these, 890 were excluded for irrelevance. Eighty-two reports were sought for full-text assessment, and 69 were excluded due to lack of biomarker diagnostic data (*n* = 45), absence of PICF/saliva specimens (*n* = 12), animal or *in vitro* design (*n* = 7), or review/meta-analysis type (*n* = 5). Ultimately, 13 studies were included in the qualitative and quantitative synthesis (Figure 1, Table 1) [3], 10], [16], [17], [18], [19], [20], [21], [22], [23], [24], [25], [26].

**Figure 1:**
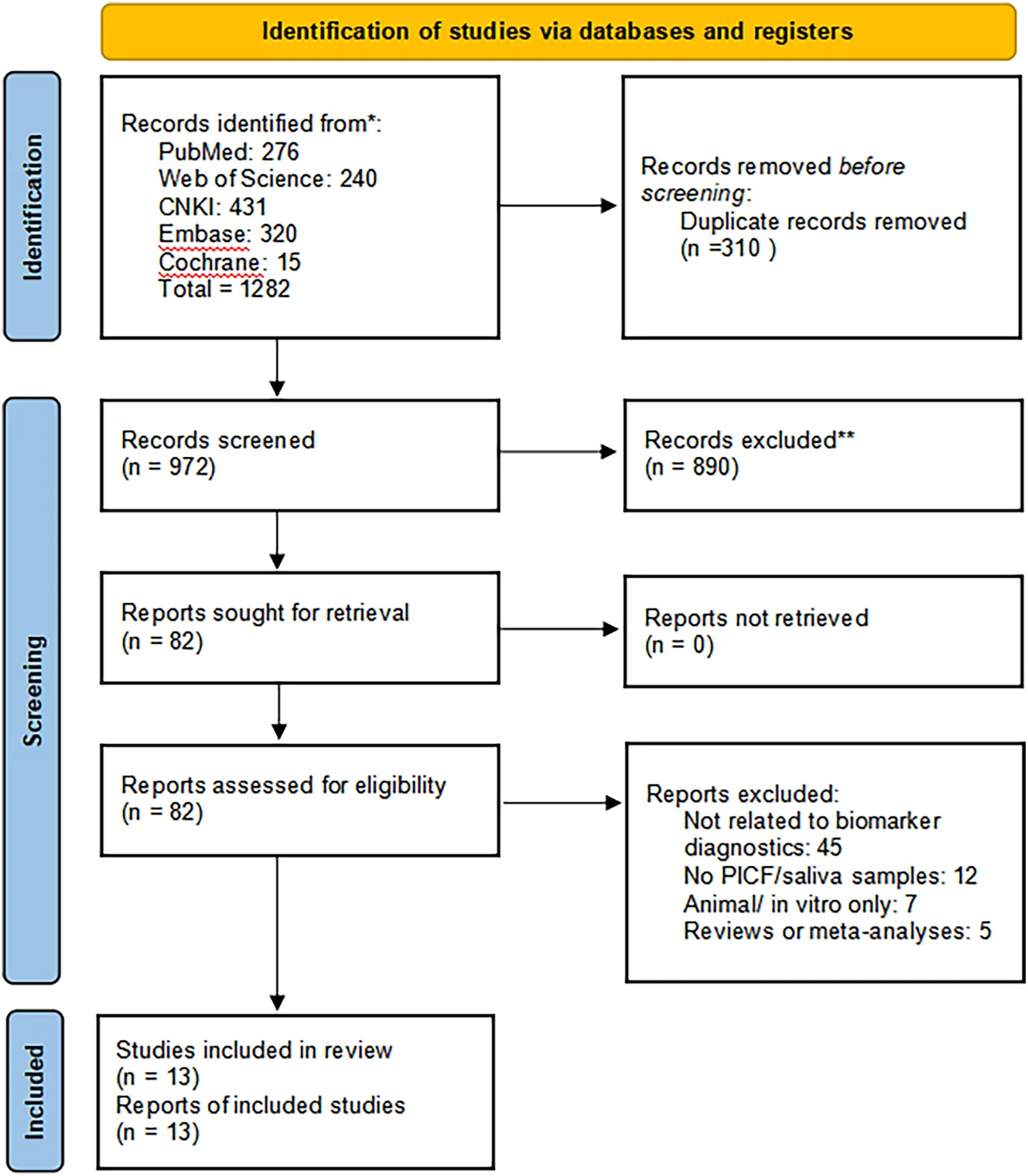
PRISMA 2020 flow diagram of study selection.

**Table 1:** Study characteristics.

First author (year)	Country/region	Sample size (PI/control)	Specimen	Assay method	Main biomarkers	Diagnostic criteria	Analysis unit
Zhang (2005) [16]	China	PI = 8, control = 23	PICF	ELISA	IL-6	PD > 4 mm + GI ≥ 1 + bone loss > 20 %	Implant
Saxena (2025) [3]	India	PI = 20, control = 20	PICF, GCF	ELISA	IL-1β, IL-6	PD ≥ 4 mm, bone loss, thread exposure	Patient
Yaghobee (2013) [17]	Iran	PI = 41 implants, control = 41 teeth	PICF, GCF	ELISA	IL-1β	PD ≥ 4 mm, BOP+, bone loss	Site
Al-Askar (2018) [18]	Saudi Arabia	PI = 74, control = 97	Saliva	ELISA	IL-1β, IL-6	PD ≥ 5 mm, bone loss ≥ 2 mm	Patient
Abduljabbar (2019) [19]	Saudi Arabia	PI = 21, control = 25	Saliva	ELISA	IL-1β, IL-6, TNF-α	PD ≥ 4 mm, BOP ≥ 30 %, CBL ≥ 3 mm	Patient
Yaghobee (2014) [20]	Iran	PI = 8, control = 8	PICF	ELISA	IL-1β, IL-6	PD ≥ 6 mm, bone loss	Implant
Gao (2018) [21]	China (Xinjiang)	PI = 40, control = 40	PICF	Multiplex immunoassay	IL-1β, MMP-8, IL-17A	BOP + PD increase + bone loss	Patient
Luo (2011) [22]	China	PI = 20, control = 20	PICF	ELISA, Western blot	IL-1β, IL-6, IL-8, TNF-α, HMGB1	PD ≥ 5 mm, bone loss	Patient
Gündoğar (2021) [23]	Turkey	PI = 14, control = 13	PICF	ELISA	IL-1β, TNF-α	2017 EFP/AAP criteria	Implant
Wang (2022) [24]	China	PI = 45, control = 98	PICF	ELISA	SIRT1, IL-1β, IL-6, TNF-α, CRP	2017 World Workshop	Patient
Chen (2020) [25]	China	PI = 12, control = 12	PICF	ELISA	IL-1β, IL-6, TNF-α	PD ≥ 6 mm, BOP, bone loss	Implant
Bhukal (2025) [26]	India	PI = 32, control = 32	PICF	ELISA	IL-6, IL-10, TNF-α	PD ≥ 4 mm, BOP+, bone loss ≥ 2 mm	Patient
Yang (2024) [10]	China	T2DM + CP = 147, CP = 128, control = 121	PICF	ELISA	SIRT1, SIRT6	CP + T2DM diagnostic	Patient

### 3.2 Risk of bias assessment

Thirteen eligible studies were included, covering populations from China, India, Iran, Saudi Arabia, and Turkey. Most studies investigated inflammatory cytokines (IL-1β, IL-6, TNF-α), while two focused on oxidative stress regulators (SIRT1). Sample sizes ranged from fewer than 20 implants to over 200 patients. Risk of bias assessment indicated that the majority of studies were of low risk, although several earlier studies (Zhang [16]; Yaghobee [17], 20]; Chen [25]) were considered moderate risk due to limited sample sizes and implant-level analyses (Figure 2, Table 2).

**Figure 2:**
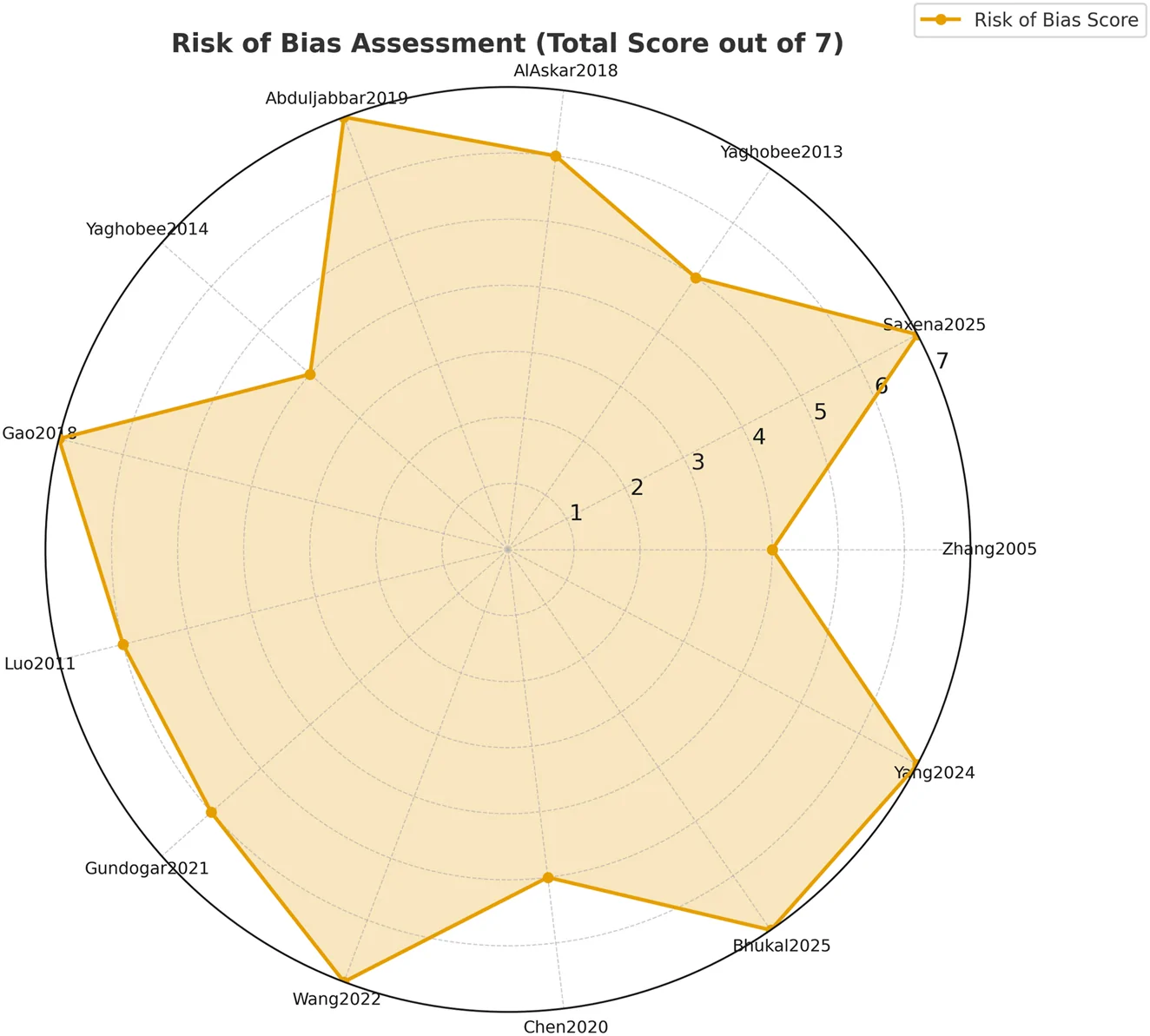
Risk of bias assessment of included studies.

**Table 2:** Risk of bias assessment.

First author (year)	Representativeness	Sample size adequate	Exposure measurement	Confounders addressed	Outcome assessment	Statistical analysis	Total score (*n*/7)	Risk level
Zhang (2005) [16]	Ture	Flase (*n* = 8 PI)	Ture	Flase	Ture	Ture	4	Moderate risk
Saxena (2025) [3]	Ture	Ture	Ture	Ture	Ture	Ture	7	Low risk
Yaghobee (2013) [17]	Ture	Flase (implant-level)	Ture	Flase	Ture	Ture	5	Moderate risk
Al-Askar (2018) [18]	Ture	Ture	Ture	Flase	Ture	Ture	6	Low risk
Abduljabbar (2019) [19]	Ture	Ture	Ture	Ture	Ture	Ture	7	Low risk
Yaghobee (2014) [20]	Ture	Flase (*n* = 8 PI)	Ture	Flase	Ture	Ture	4	Moderate risk
Gao (2018) [21]	Ture	Ture	Ture	Ture	Ture	Ture	7	Low risk
Luo (2011) [22]	Ture	Ture	Ture	Flase	Ture	Ture	6	Low risk
Gündoğar (2021) [23]	Ture	Ture	Ture	Flase	Ture	Ture	6	Low risk
Wang (2022) [24]	Ture	Ture	Ture	Ture	Ture	Ture	7	Low risk
Chen (2020) [25]	Ture	Flase (*n* = 12 PI)	Ture	Flase	Ture	Ture	5	Moderate risk
Bhukal (2025) [26]	Ture	Ture	Ture	Ture	Ture	Ture	7	Low risk
Yang (2024) [10]	Ture	Ture	Ture	Ture	Ture	Ture	7	Low risk

### 3.3 Pooled effects of inflammatory biomarkers

IL-1β: Seven studies (145 PI vs 213 controls) provided quantitative data on IL-1β in PICF. The pooled standardized mean difference (SMD) was 2.09 (95 % CI: 1.76–2.42; *p* < 0.0001) under a random-effects model, with no significant heterogeneity (*I* ^2^ = 0.0 %). The 95 % prediction interval (PI) was 1.46–2.72, confirming consistent elevation in PI patients (Figure 3).

**Figure 3:**
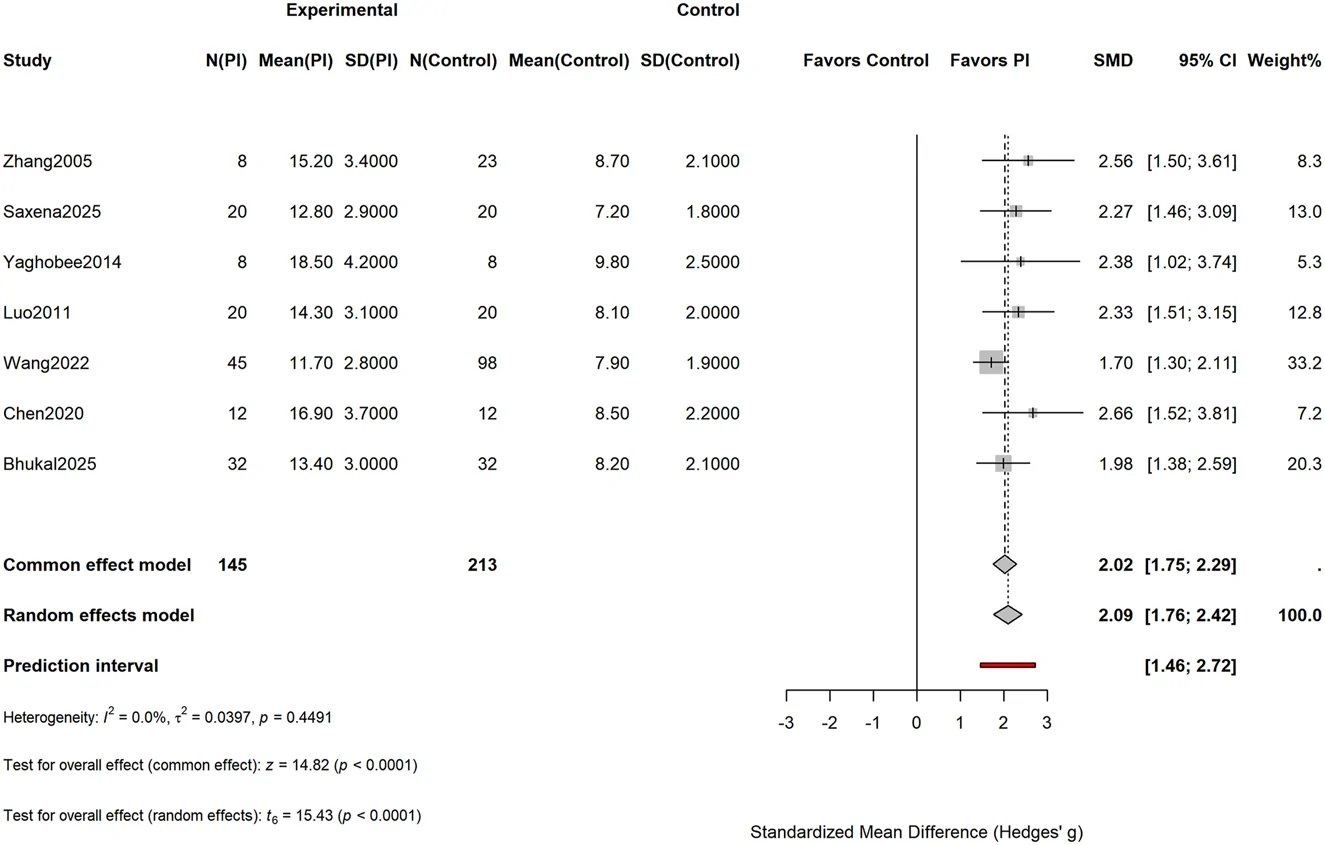
Forest plot of pooled SMD for IL-1β levels in peri-implantitis versus healthy controls.

IL-6: Nine studies (274 PI vs 349 controls) reported IL-6 concentrations. The pooled SMD was 3.19 (95 % CI: 3.05–3.33; *p* < 0.0001) with negligible heterogeneity (*I* ^2^ = 0.0 %). The prediction interval was 2.90–3.47, indicating a consistently positive between-group difference across the included studies (Figure 4, Table 2).

**Figure 4:**
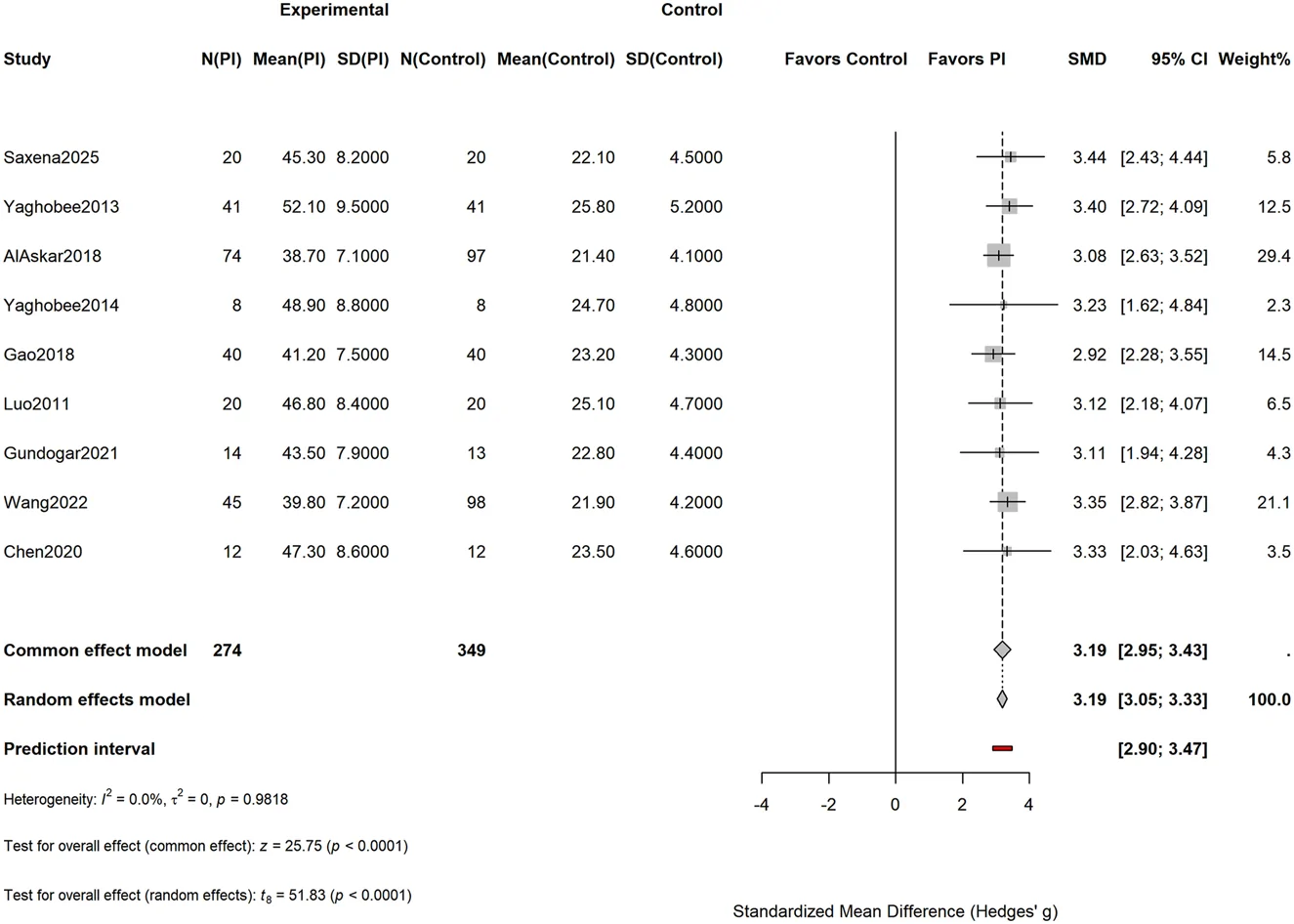
Forest plot of pooled standardized mean difference (SMD) for IL-6 levels in peri-implantitis versus healthy controls.

TNF-α: Six studies (144 PI vs 200 controls) were included. The pooled SMD was 3.06 (95 % CI: 2.85–3.28; *p* < 0.0001) with low heterogeneity (*I* ^2^ = 0.0 %). The 95 % PI was 2.64–3.48, indicating a consistently higher TNF-α level in PI groups than in controls across the included studies (Figure 5).

**Figure 5:**
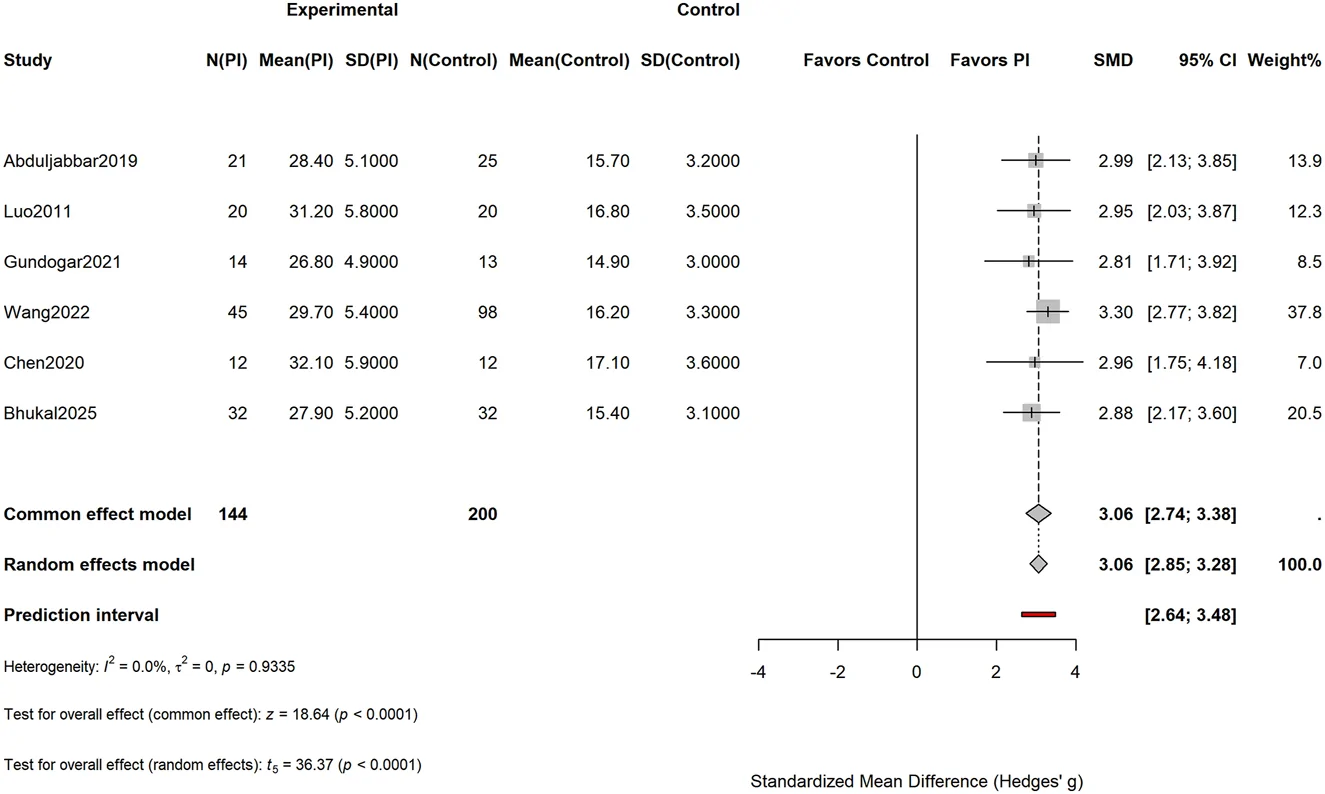
Forest plot of pooled SMD for TNF-α levels in peri-implantitis versus healthy controls.

Overall, the three inflammatory cytokines demonstrated large and consistent between-group differences, supporting their role as reproducible inflammatory correlates of peri-implantitis rather than definitive standalone diagnostic markers. The very low *I* ^2^ estimates were supported by Cochran Q statistics below their corresponding degrees of freedom and *τ* ^2^ estimates of 0.00 in the primary cytokine models, indicating that the observed dispersion of study-specific SMDs was compatible with within-study sampling variation. The large pooled SMDs therefore reflected consistently positive and non-overlapping biomarker distributions between peri-implantitis and control groups rather than a transformation artifact.

### 3.4 Subgroup and sensitivity analyses

Subgroup analyses confirmed the robustness of the pooled effect sizes for inflammatory cytokine concentrations (IL-1β, IL-6, TNF-α) in peri-implantitis versus controls (Figure 6).

**Figure 6:**
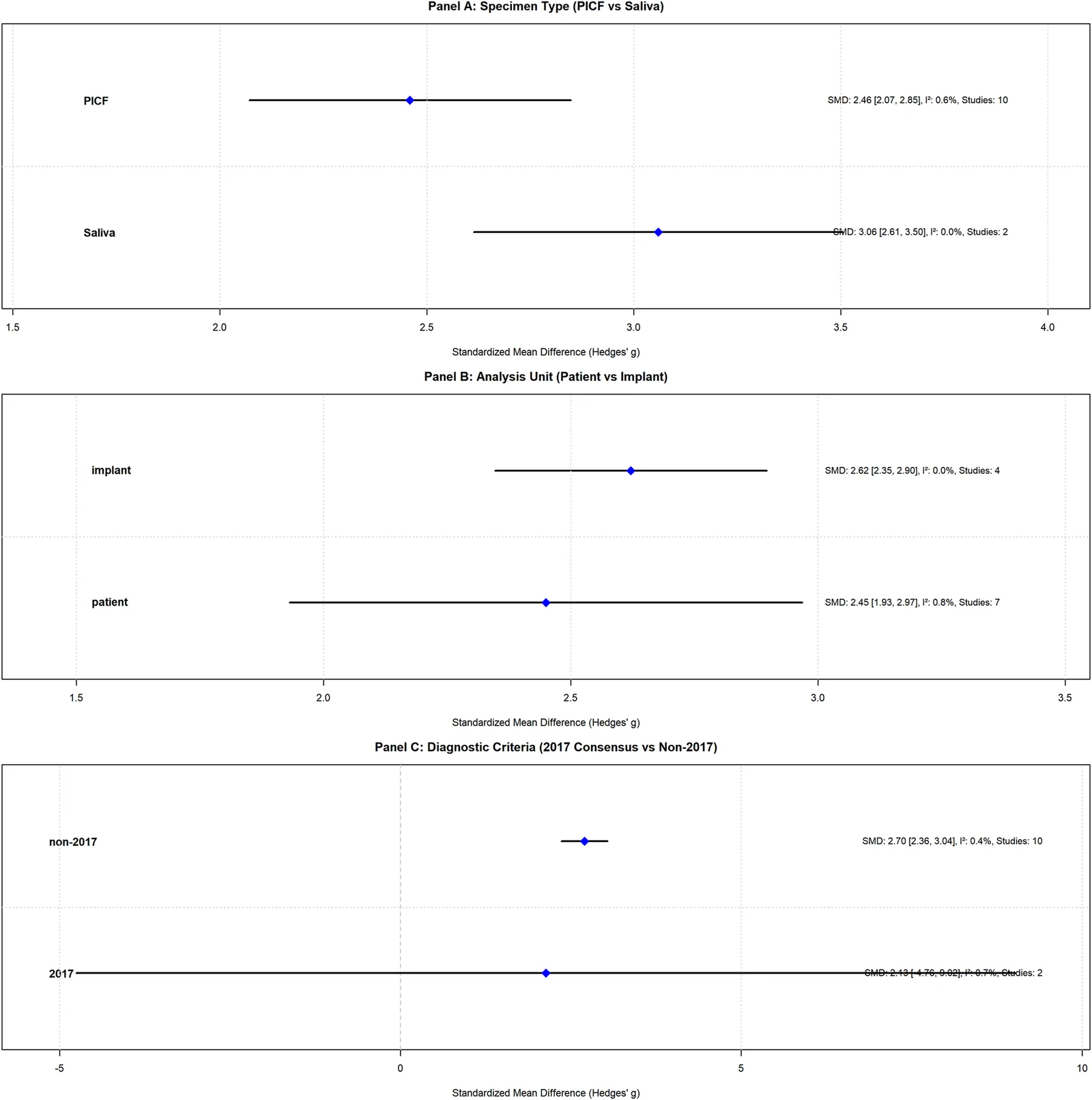
Subgroup analyses of inflammatory biomarkers stratified by specimen type (PICF vs saliva), analysis unit (patient vs implant), and diagnostic criteria (2017 consensus vs non-consensus).

Specimen type: Significant differences were observed for both peri-implant crevicular fluid (PICF; SMD = 2.46, 95 % CI: 2.07–2.85) and saliva (SMD = 3.06, 95 % CI: 2.61–3.50), with negligible heterogeneity.

Analysis unit: Implant-level studies (SMD = 2.62; 95 % CI: 2.35–2.90) and patient-level studies (SMD = 2.45; 95 % CI: 1.93–2.97) yielded consistent results.

Diagnostic criteria: Studies using non-2017 diagnostic criteria demonstrated a robust pooled effect (SMD = 2.70, 95 % CI: 2.36–3.04). Studies applying the 2017 consensus criteria also showed a positive association (SMD = 2.43), although the confidence interval was wide (95 % CI: −4.76 to 9.62), reflecting limited sample size.

These findings demonstrate that the biomarker effects are not materially influenced by sample type, analysis level, or diagnostic criteria.

### 3.5 Heterogeneity and influence diagnostics

Heterogeneity across studies was generally negligible (*I* ^2^ = 0 %). Baujat and influence diagnostics identified a few studies (Chen [25]; Wang [24]) as contributing relatively more to heterogeneity, but no extreme outliers were detected. Leave-one-out analyses confirmed the stability of pooled results (Figure 7). The absence of measurable between-study heterogeneity was further examined by comparing study-level SMDs and standard errors across the three cytokine models. For IL-1β, IL-6, and TNF-α, all study-specific effects were positive and aligned in magnitude, and no individual study simultaneously showed a discordant direction and a high influence statistic. This pattern explains why large summary effects could coexist with *I* ^2^ = 0.0 % in the present dataset.

**Figure 7:**
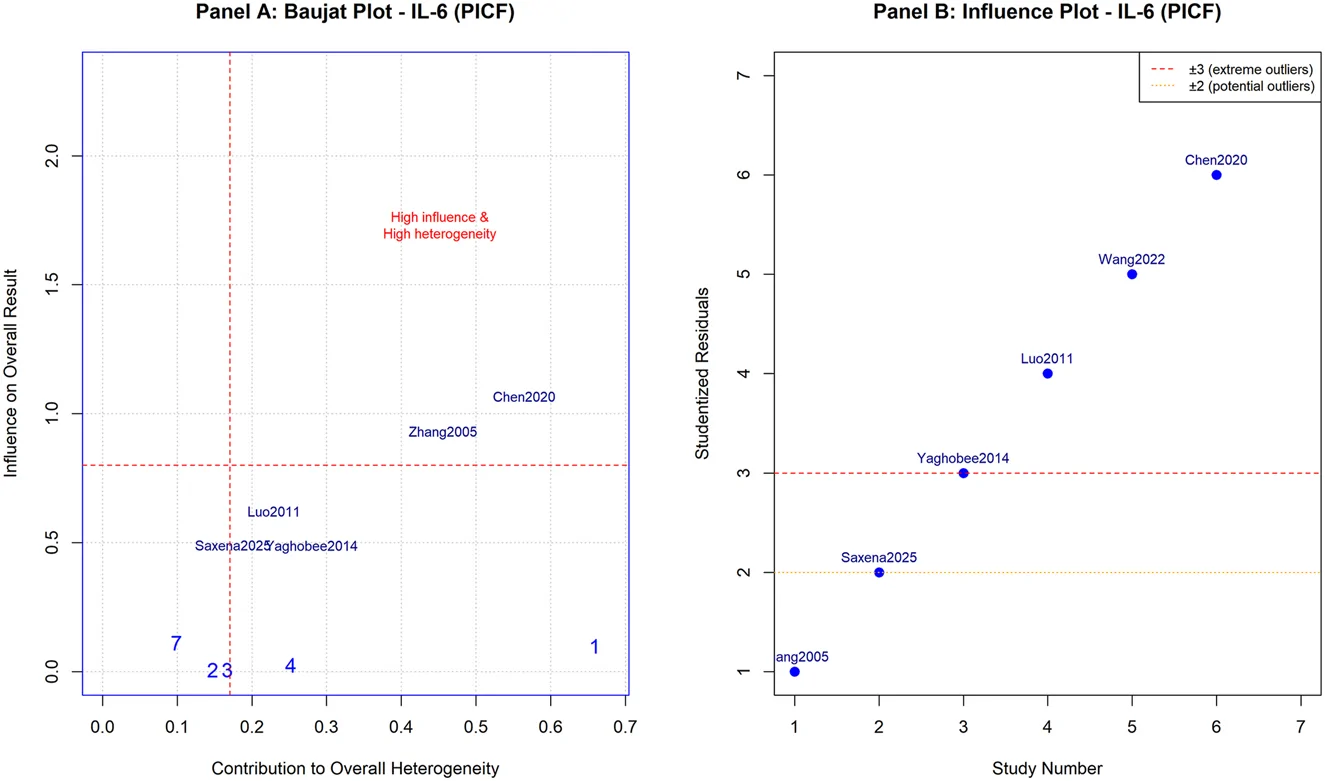
Leave-one-out sensitivity analyses and Baujat influence diagnostics for included studies.

### 3.6 Diagnostic performance of SIRT1

Two studies evaluated the discriminatory performance of SIRT1 (Figure 8). Because of the scant sample, HSROC curves and optimal cutpoints could not be generated, underscoring the preliminary nature of these diagnostic findings.

**Figure 8:**
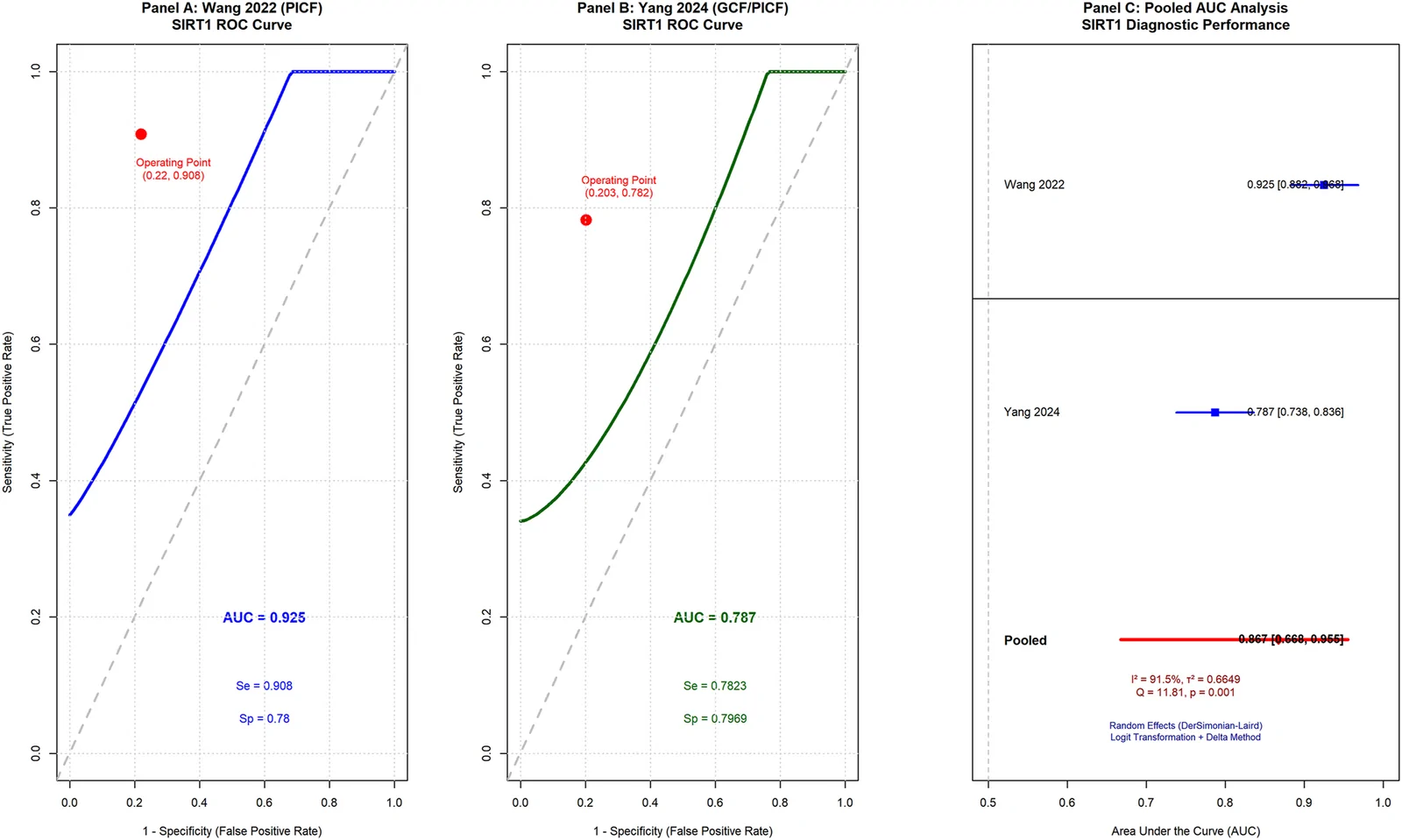
Pooled diagnostic performance of SIRT1: summary ROC curves and forest plots of area under the curve (AUC).

Wang [24] (PICF): AUC = 0.925, sensitivity = 0.908, specificity = 0.780.

Yang [10] (GCF/PICF): AUC = 0.787, sensitivity = 0.782, specificity = 0.797.

Pooled AUC (logit random-effects): 0.867 (95 % CI: 0.660–0.965).

These results suggest that SIRT1 may have promising discriminatory potential, although the evidence remains limited to two studies and is insufficient for firm diagnostic inferences.

### 3.7 Meta-regression analyses

Meta-regression explored study-level covariates for effect sizes of biomarker concentration differences (SMD) between peri-implantitis and control groups (Figures 9 and 10).

**Figure 9:**
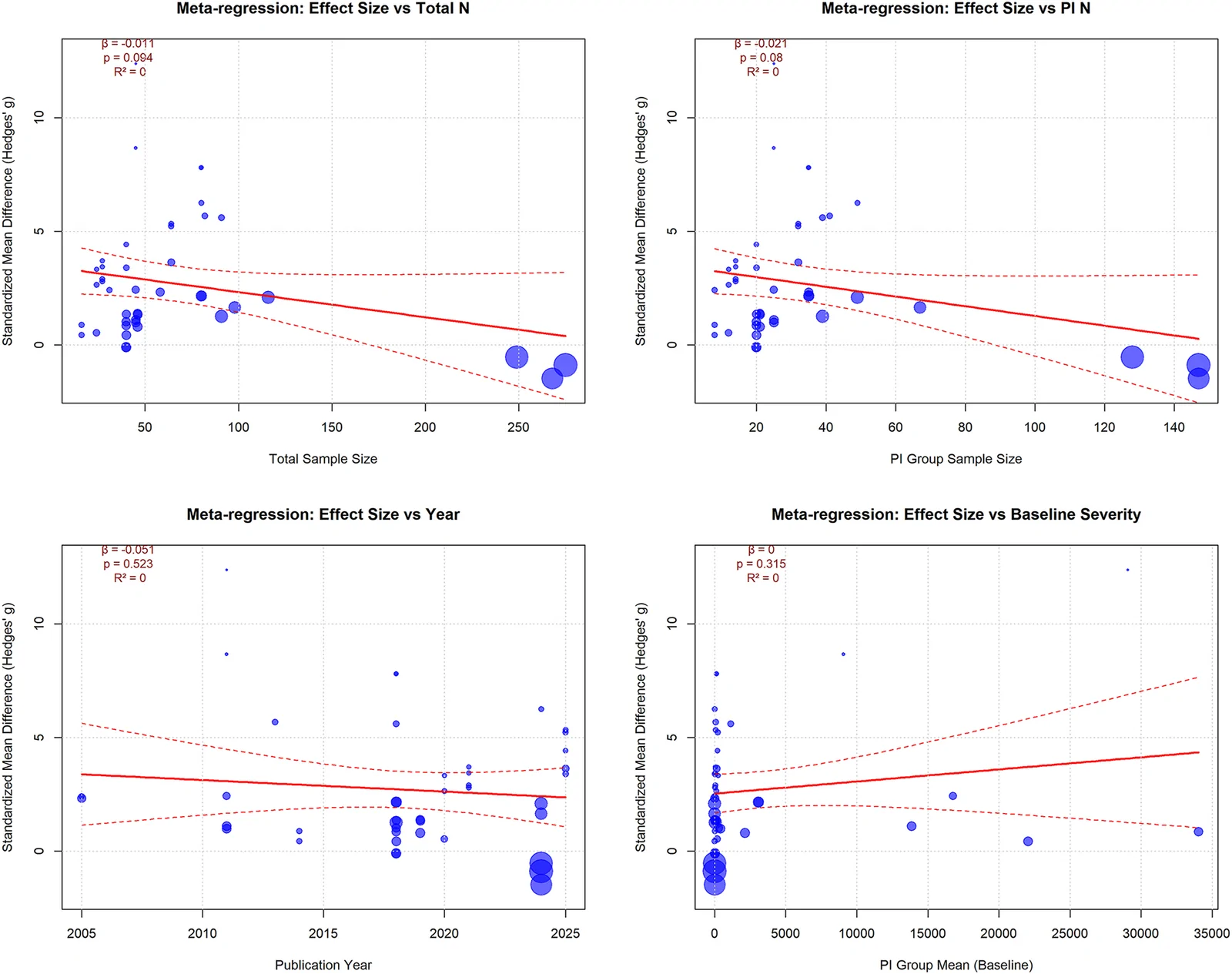
Bubble plot of meta-regression exploring covariates influencing effect sizes.

**Figure 10:**
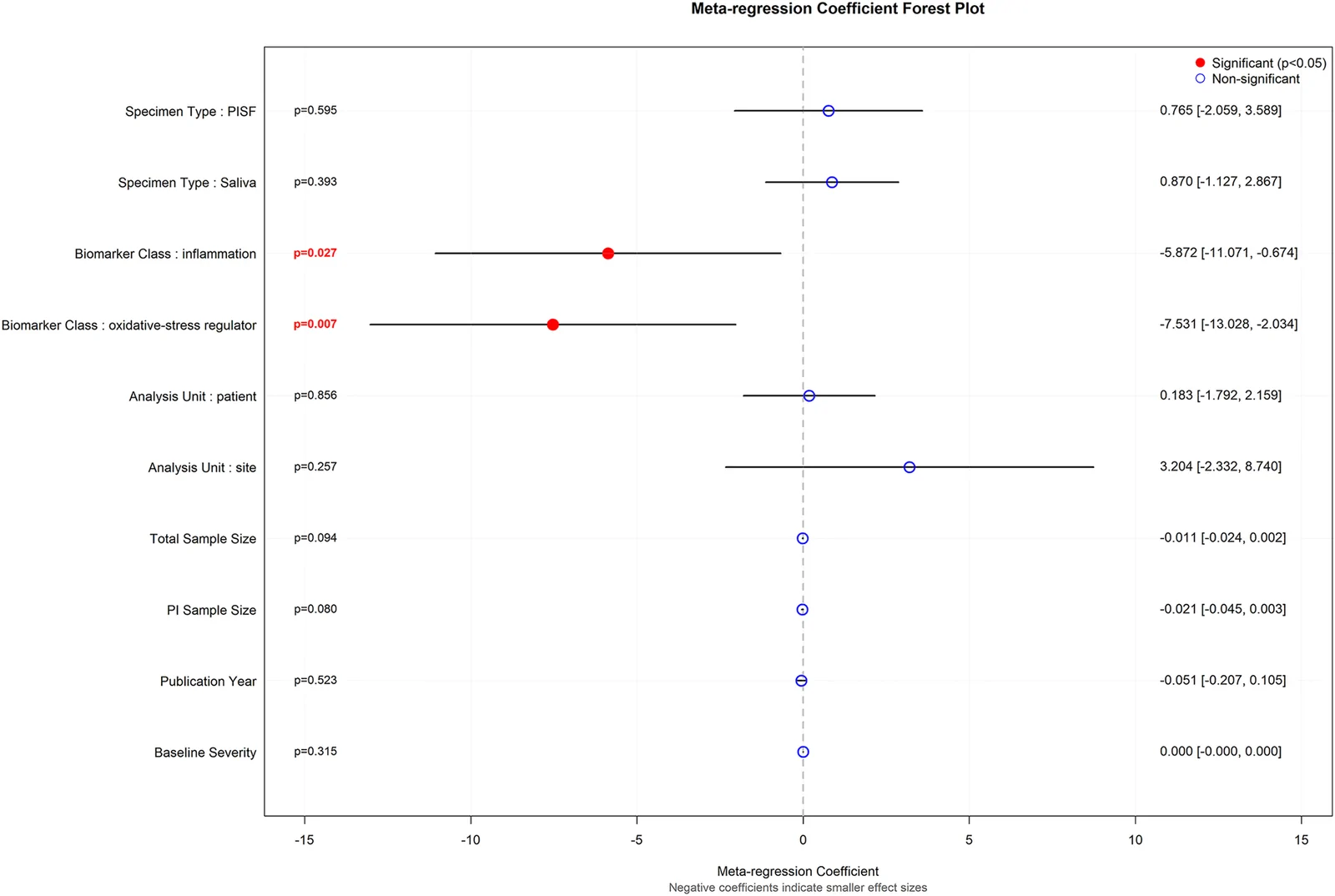
Coefficient forest plot from meta-regression showing biomarker class as a significant moderator.

Bubble plots showed no significant associations between effect size and sample size, PI sample size, publication year, or baseline severity (Figure 9).

Coefficient forest plots (Figure 10) demonstrated that biomarker class significantly influenced effect size: inflammatory markers had higher pooled effects, whereas oxidative-stress regulators (SIRT1) were associated with smaller effect sizes (*β* = −7.53, *p* = 0.007).

Specimen type and analysis unit did not significantly modify pooled results.

### 3.8 Publication bias assessment

The possibility of publication bias was examined using funnel plots, contour-enhanced plots, radial plots, and trim-and-fill analyses.

For IL-1β, the funnel plot appeared roughly symmetrical (Figure 11). Contour-enhanced plots and radial plots showed no significant asymmetry. The overall effect was robust.

**Figure 11:**
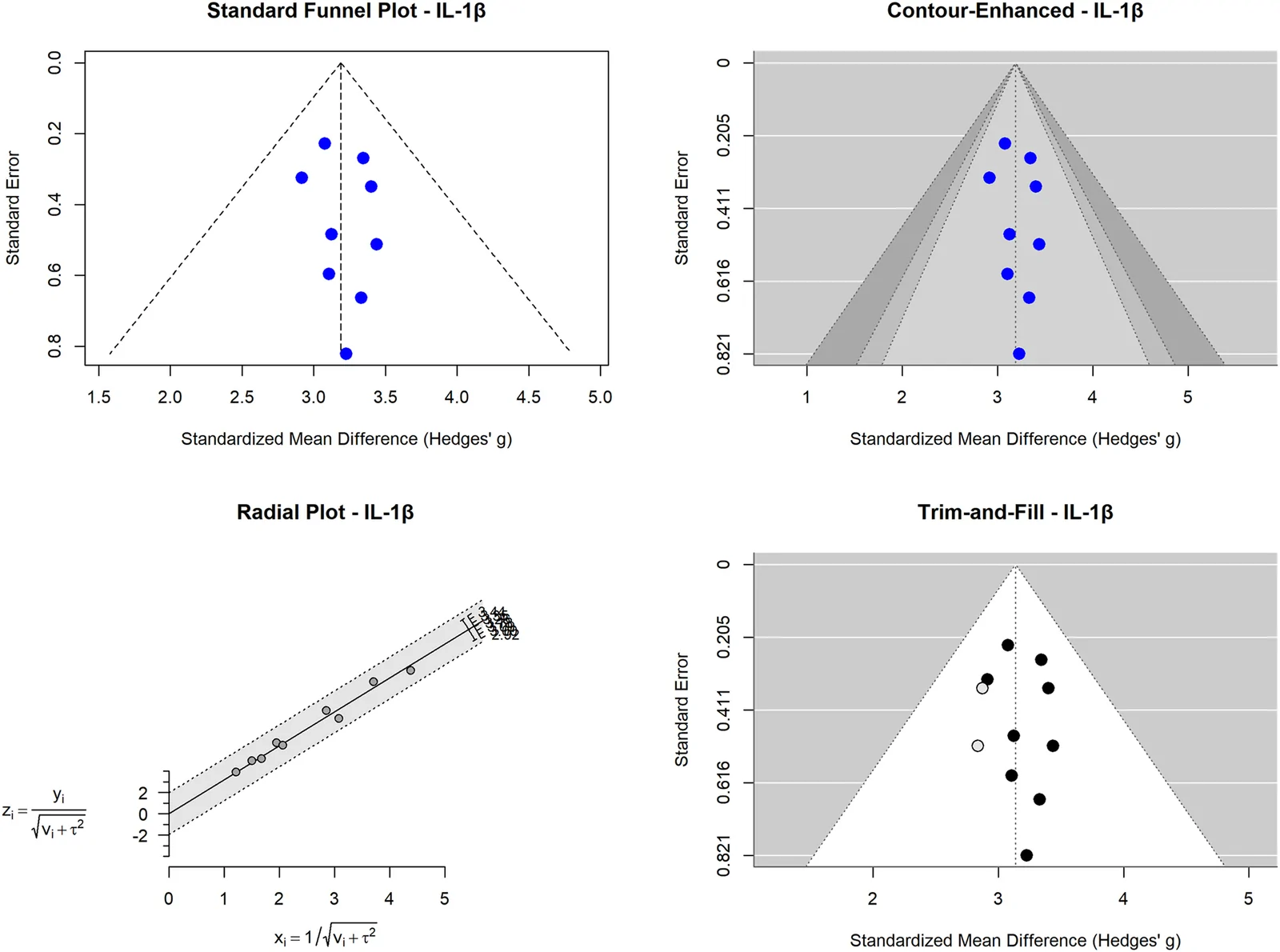
Funnel plot and contour-enhanced plot assessing publication bias for IL-1β.

For IL-6, visual inspection of the funnel plot revealed some asymmetry, with relatively few small studies reporting small effect sizes (Figure 12). Trim-and-fill analysis revealed four missing studies on the left side of the funnel. After imputation, the adjusted mean difference (SMD) decreased but remained statistically significant.

**Figure 12:**
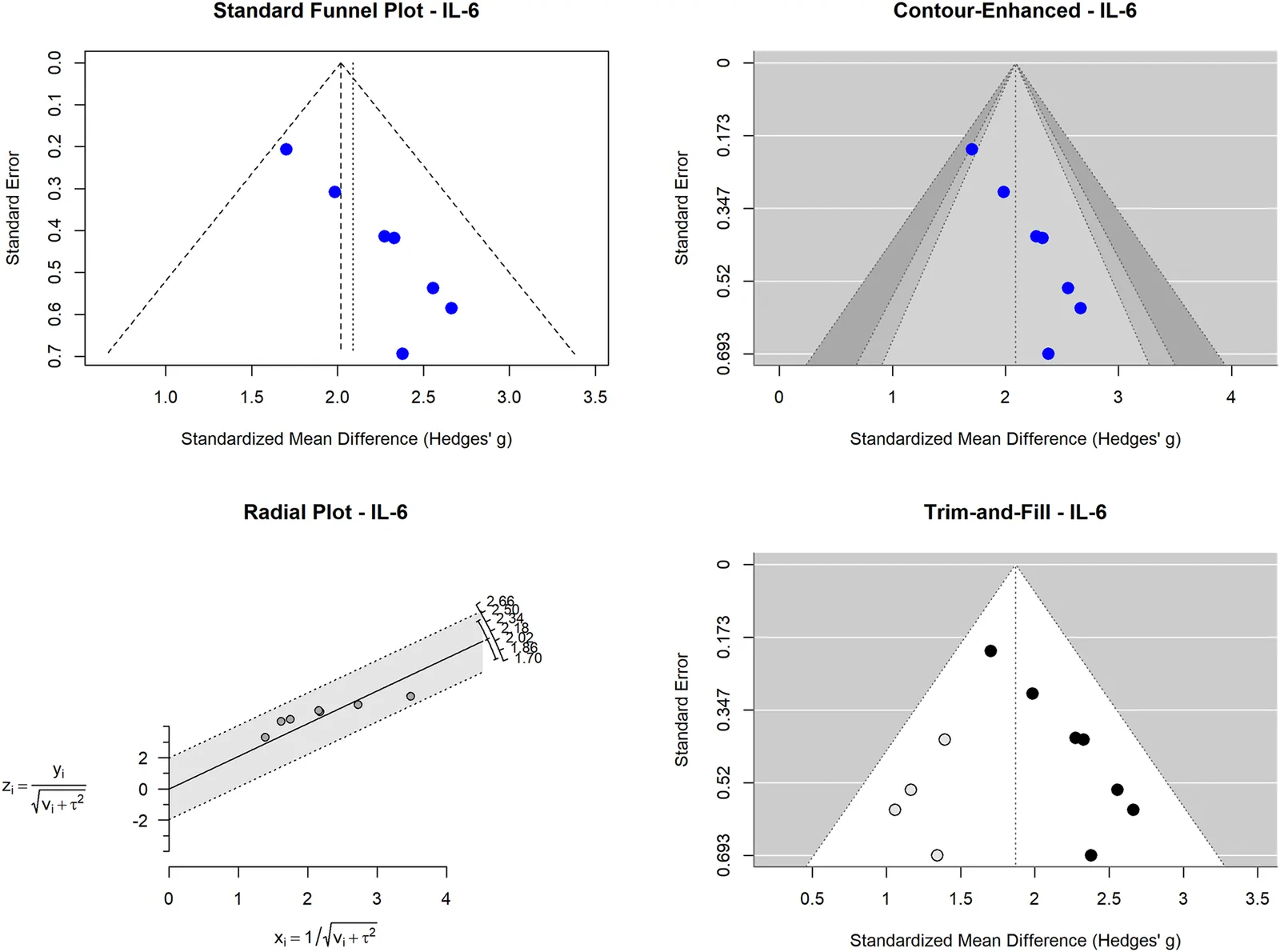
Funnel plot and trim-and-fill analysis for IL-6.

For TNF-α, the funnel plot appeared nearly symmetrical, and contour-enhanced plots and radial plots showed no significant bias (Figure 13). Trim-and-fill analysis imputed two hypothetical studies, with a negligible effect on the pooled SMD. Therefore, no publication bias was observed for TNF-α. Overall, these analyses showed that, despite a slight asymmetry (IL-6), the overall pooled estimates for IL-1β, IL-6, and TNF-α were relatively stable, and the conclusions of the meta-analysis were not significantly altered by potential publication bias.

**Figure 13:**
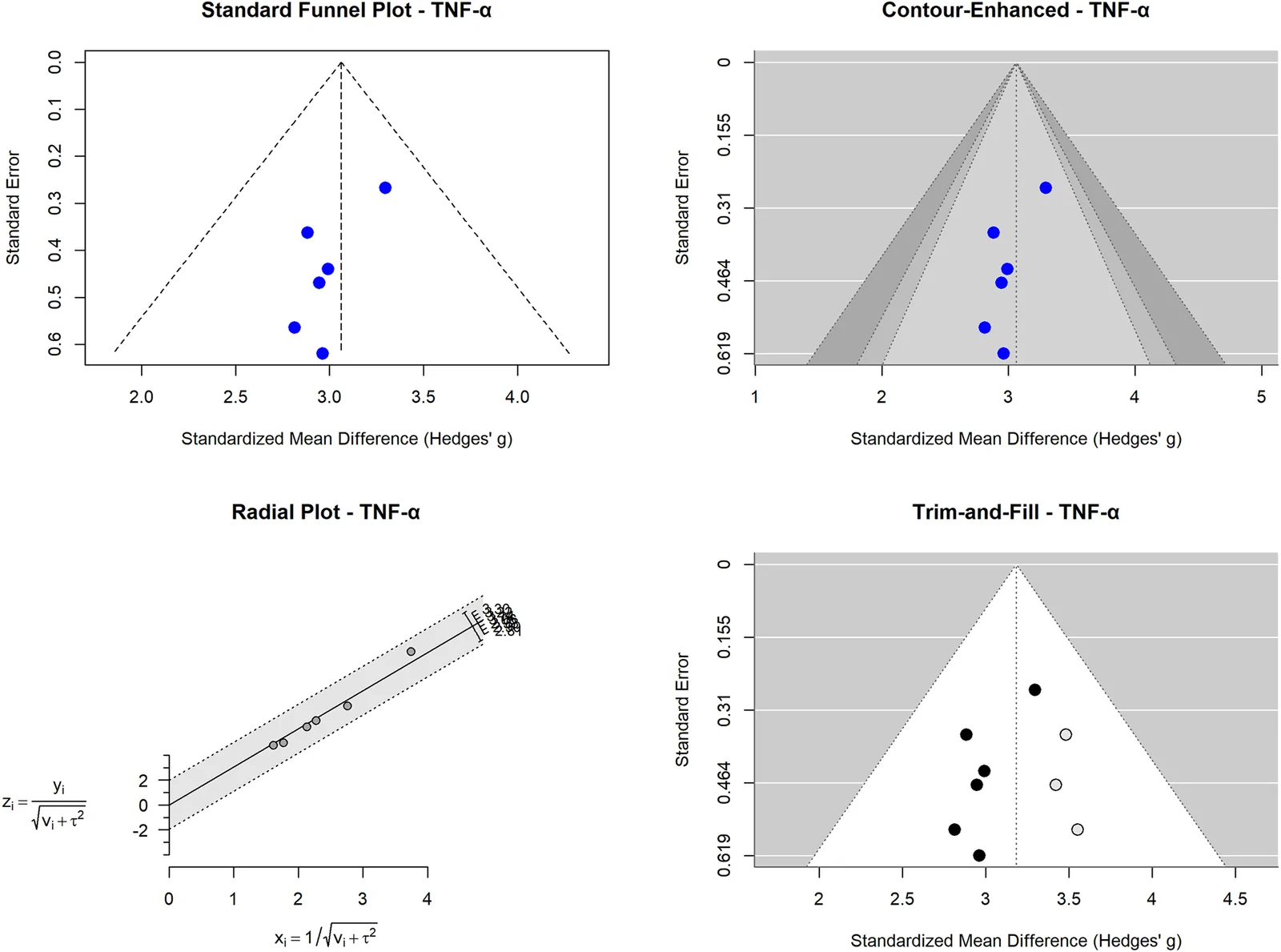
Funnel plot and contour-enhanced plot assessing publication bias for TNF-α.

### 3.9 Sensitivity analysis

Leave-one-out analyses did not materially alter the pooled direction or statistical significance of the estimates for IL-1β, IL-6, and TNF-α. Additional analyses excluding small studies produced similar pooled estimates for IL-1β (SMD = 1.92, 95 % CI: 1.63–2.21; *I* ^2^ = 0.0 %), IL-6 (SMD = 3.19, 95 % CI: 2.93–3.44; *I* ^2^ = 0.0 %), and TNF-α (SMD = 3.09, 95 % CI: 2.75–3.44; *I* ^2^ = 0.0 %). When the highest-effect study was omitted for each cytokine, the estimates remained materially unchanged for IL-1β (SMD = 1.99, 95 % CI: 1.71–2.26; *I* ^2^ = 0.0 %), IL-6 (SMD = 3.17, 95 % CI: 2.93–3.42; *I* ^2^ = 0.0 %), and TNF-α (SMD = 2.92, 95 % CI: 2.52–3.32; *I* ^2^ = 0.0 %). These findings indicate that the conclusions were not driven by small studies or by a single high-effect study. Detailed numerical results for the primary and additional exclusion analyses are summarized in Supplementary Table S1, and a comparative sensitivity-analysis plot is presented in Supplementary Figure S1.

## 4 Discussion

### 4.1 Principal findings

Across 13 eligible studies from five Asian countries, we found significantly higher levels of inflammatory cytokines in oral fluids of patients with peri-implantitis (PI). Random-effects models showed pooled SMDs of 3.19 for IL-6, 3.06 for TNF-α, and 2.09 for IL-1β, with negligible heterogeneity and narrow 95 % prediction intervals (Figures 3–5). Subgroup analyses demonstrated robustness across specimen type (PICF vs saliva), analysis unit (patient vs implant), and diagnostic criteria (2017 consensus vs non-2017) (Figure 6). Publication-bias diagnostics suggested minor asymmetry for IL-6 but little impact on the pooled effects, while IL-1β and TNF-α were largely symmetrical (Figure 7 and funnel-plot suite). For SIRT1, two studies yielded a pooled AUC of approximately 0.88 after logit transformation and random-effects modeling, suggesting potentially favorable discriminatory performance; however, the evidence base remains limited and preliminary (Figure 8). Meta-regression highlighted biomarker class as a significant source of variability – inflammatory markers producing larger effects than oxidative-stress regulators (Figures 9 and 10). Because these summary effects were derived from raw mean and standard-deviation contrasts rather than log-transformed cytokine values, their magnitude should be interpreted as standardized between-group separation within the included datasets, not as a fold-change in biomarker concentration. The concordant direction of the individual studies and the *τ* ^2^ = 0.00 estimates explain the negligible *I* ^2^ values despite large pooled SMDs.

More broadly, recent peri-implant biomarker literature has increasingly moved from single-cytokine comparisons toward multimarker and pathway-oriented frameworks, including oxidative-stress markers, resilience/resistance pathways, and metabolomic profiling. This broader perspective is relevant because peri-implantitis is unlikely to be adequately characterized by one inflammatory analyte alone. Instead, currently available evidence suggests a layered host-response model in which classical cytokines primarily reflect inflammatory burden, whereas regulatory markers such as SIRT1 may provide complementary information, although evidence for these newer markers remains limited.

### 4.2 Comparison with prior literature

Our results align with and extend earlier narrative and systematic reviews showing that PICF harbors a pro-inflammatory signature in PI, including elevations in IL-1β, IL-6, TNF-α, IL-8, and MMP-8 [16], [17], [18], [19]. By pooling only Asian cohorts and separating PICF from saliva, we show that these signals are quantitatively large and remarkably homogeneous, suggesting that specimen type and clinical definitions contribute little to between-study dispersion once core collection/assay practices are standardized. The diagnostic literature on SIRT1 remains nascent; preclinical and periodontal studies support a homeostatic, anti-inflammatory role for SIRT1 via NF-κB and NLRP3 regulation, osteogenic differentiation, and redox control [20], 21]. The currently available clinical evidence is consistent with a possible host-protective role of SIRT1, but the pooled AUC should be interpreted cautiously given the very limited number of eligible studies.

### 4.3 Biological plausibility

The magnitude and stability of IL-6/IL-1β/TNF-α effects are biologically coherent with microbe-driven innate and adaptive immune activation in peri-implant tissues, where these cytokines promote osteoclastogenesis, matrix degradation, and soft-tissue inflammation, reinforcing the transition from mucositis to bone-involving lesions [22], 23]. Conversely, SIRT1, a NAD^+^-dependent deacetylase, dampens NF-κB–mediated transcription, restrains NLRP3 inflammasome activation, and supports periodontal ligament/osteoblast lineage stability [10], 24]; lower SIRT1 may plausibly reflect inflammatory burden and impaired tissue homeostasis; however, this biological interpretation remains hypothesis-supportive rather than clinically established in peri-implantitis, given the limited number of eligible studies.

### 4.4 Clinical and translational implications

Three implications emerge. First, the large pooled differences in IL-6, TNF-α, and IL-1β suggest that these cytokines may have adjunctive relevance in peri-implantitis assessment, particularly as inflammatory correlates alongside clinical and radiographic findings. Second, SIRT1 shows potentially informative discriminatory performance and mechanistic complementarity; however, the current evidence is limited to two studies and remains insufficient for firm diagnostic interpretation. Third, the PICF versus saliva results suggest that saliva may also reflect peri-implant inflammatory status, although its practical applicability for screening or triage requires prospective validation and standardized protocols [25], [26], [27], [28]. Overall, these biomarkers should currently be viewed as investigational adjuncts rather than established standalone diagnostic tools.

### 4.5 Heterogeneity, risk of bias, and publication bias

Heterogeneity was minimal across the primary cytokine analyses (*I* ^2^ = 0 %). Although this pattern may appear unusual given the large pooled SMDs, several features of the included literature may explain it: most studies compared peri-implantitis with clinically healthy controls within Asian adult populations, evaluated broadly similar inflammatory biomarkers, and showed highly concordant effect directions. Thus, the low statistical heterogeneity should not be interpreted as proof of universal transportability, but rather as reflecting a relatively homogeneous clinical contrast within the currently available evidence base (Figure 7). Most studies were low risk by JBI/NOS; four earlier reports were moderate risk largely due to small sample sizes and implant-level analysis. Funnel-plot suites indicated minor small-study effects for IL-6; trim-and-fill moved pooled estimates only modestly, suggesting conclusions are robust to potential publication bias. The additional exclusion analyses strengthened this interpretation: removing small studies and removing the highest-effect study for each cytokine did not change the direction, statistical significance, or approximate magnitude of the pooled effects. These checks support the robustness of the cytokine findings while acknowledging that the number of eligible Asian studies remains limited.

### 4.6 Strengths and advances

Methodologically, we (i) distinguished PICF from saliva and used prediction intervals, (ii) implemented logit(AUC) random-effects pooling for SIRT1, and (iii) used meta-regression to test prespecified moderators (biomarker class, assay, analysis unit, criteria), revealing biomarker class as a key driver of effect magnitude. Subgroup stability across 2017 consensus versus non-consensus criteria provides reassurance of clinical generalizability within Asian cohorts.

### 4.7 Limitations and future directions

Several caveats remain. First, although supplementary protocol registration was initiated during the revision process, the review was not prospectively registered before the literature search and data extraction were conducted, which may still limit methodological transparency. Second, because most included studies were cross-sectional comparisons rather than formal diagnostic-accuracy studies, the present findings should be interpreted primarily as evidence of between-group biomarker differences and possible discriminatory potential, not as proof of established diagnostic performance. Third, residual confounding (e.g., smoking, diabetes status, implant loading time, prosthetic design, maintenance) was variably reported or adjusted. Fourth, pre-analytical variability (paper-strip dwell time, pooling per site vs per implant, storage conditions) and assay heterogeneity (ELISA kits, multiplex calibration) can shift absolute concentrations, though SMDs mitigate scale differences. Fifth, the SIRT1 evidence comprises only two diagnostic studies; therefore, the pooled AUC should be viewed as preliminary, exploratory, and insufficient for definitive inference. Finally, unit-of-analysis issues persist in older implant-level studies; our sensitivity and moderator analyses reduce but cannot entirely remove this bias. Beyond SIRT1, no eligible studies evaluated other oxidative or immune-regulatory markers (e.g., SIRT6, MDA, TAC, antioxidant enzymes), so inferences about these pathways are provisional. Heterogeneous peri-implantitis definitions (pre- vs post-2017 thresholds, radiographic bone-loss requirements) and site selection protocols may still introduce misclassification despite subgrouping. Most datasets originated from China and the Middle East, with South/Southeast Asia underrepresented, which limits regional generalizability even within Asia. IL-6 displayed funnel-plot asymmetry suggestive of small-study effects, reinforcing the need for pre-registered, adequately powered cohorts. No study reported actionable thresholds, decision-curve analyses, or cost-effectiveness, leaving clinical utility undetermined and dependent on future prospective validation. Future studies should be prospective, multi-center, and pre-registered, apply uniform 2017 consensus staging, and ensure patient-level analysis to avoid inflated sample sizes. Core covariates (smoking, diabetes/glycemic control, implant vintage and loading time, prosthesis type, supportive maintenance frequency, antibiotic/anti-inflammatory use) should be collected and adjusted to reduce residual confounding, particularly for saliva markers. Standardized pre-analytical workflows (sampling time of day, fasting status, unstimulated vs stimulated saliva, paper-point dwell time/number of sites, storage temperature, hemolysis control) and assay calibration are prerequisites for threshold transportability. Prospective designs should pair biomarker trajectories with clinical/radiographic outcomes (bone-level changes, suppuration/BOP resolution) and treatment response to enable prognostic modeling. Economic and implementation endpoints (cost per test, chairside feasibility, decision-curve analysis) are needed to judge clinical utility in diverse Asian healthcare settings [29], [30], [31], [32].

We recommend large, prospective, multi-center cohorts with standardized PICF protocols (sampling time, site number, storage, blinding), harmonized 2017 consensus staging, and co-reporting of confounders to facilitate adjusted analyses. For diagnostics, future work should: (i) generate HSROC-ready datasets for SIRT1 (and other regulatory markers), (ii) test multi-marker panels (e.g., SIRT1 + IL-6 + MMP-8) against single-analyte baselines, (iii) evaluate threshold transportability across laboratories, and (iv) assess cost-effectiveness and point-of-care feasibility. Integrating omics-driven discovery with explainable machine-learning models may yield calibrated risk scores that improve early detection and guide maintenance protocols. In addition, depositing de-identified biomarker datasets with sampling/assay metadata and pre-specified analysis plans would facilitate replication, external validation, and meta-analytic HSROC/decision-curve modeling [33], [34], [35].

## 5 Conclusions

In Asian populations, IL-6, TNF-α, and IL-1β are markedly elevated in PI with very low heterogeneity, supporting their role as reproducible inflammatory correlates of peri-implantitis in currently available Asian studies, while further validation is still needed before any diagnostic application. SIRT1 exhibits promising discriminatory potential and mechanistic plausibility, but requires validation in larger and better-designed diagnostic cohorts. Standardization of sampling and reporting, together with multi-marker modeling, represents the most practical path to clinically deployable biomarker tools for peri-implant disease. Pending standardized protocols, validated thresholds, and cost-effectiveness evidence, these biomarkers should be considered investigational adjuncts rather than standalone diagnostics for peri-implantitis.

## Supplementary Material

Supplementary Material

Supplementary Material

Supplementary Material
