# Supplementary material for: Oral-fluid inflammatory and oxidative-stress biomarkers for peri-implantitis in Asian adults: a systematic review and stratified random-effects meta-analysis
Source: Open Life Sci. 2026 Oct 16;21(1):20251360. doi: 10.1515/biol-2025-1360 (PMC13674786; doi:10.1515/biol-2025-1360)
Supplement: Supplementary file 1 [file j_biol-2025-1360_suppl_002.docx]

**Supplementary Table S1:** Additional sensitivity analyses for IL-1β, IL-6, and TNF-α in peri-implantitis versus healthy controls.

Small studies were defined as comparisons with a total sample size below 40. The high-effect analysis excluded the study with the largest absolute standardized mean difference (SMD) for each biomarker.

| **Biomarker** | **Analysis scenario** | **Pooled SMD** | **95% CI** | **I² (%)** | **Interpretation** |
| --- | --- | --- | --- | --- | --- |
| IL-1β | Primary model | 2.09 | 1.76 to 2.42 | 0.0 | Large and statistically significant elevation in peri-implantitis |
| IL-1β | Excluding small studies | 1.92 | 1.63 to 2.21 | 0.0 | Direction and magnitude remained stable |
| IL-1β | Excluding highest-effect study | 1.99 | 1.71 to 2.26 | 0.0 | Conclusion unchanged |
| IL-6 | Primary model | 3.19 | 3.05 to 3.33 | 0.0 | Large and statistically significant elevation in peri-implantitis |
| IL-6 | Excluding small studies | 3.19 | 2.93 to 3.44 | 0.0 | Direction and magnitude remained stable |
| IL-6 | Excluding highest-effect study | 3.17 | 2.93 to 3.42 | 0.0 | Conclusion unchanged |
| TNF-α | Primary model | 3.06 | 2.85 to 3.28 | 0.0 | Large and statistically significant elevation in peri-implantitis |
| TNF-α | Excluding small studies | 3.09 | 2.75 to 3.44 | 0.0 | Direction and magnitude remained stable |
| TNF-α | Excluding highest-effect study | 2.92 | 2.52 to 3.32 | 0.0 | Conclusion unchanged |

*Abbreviations: SMD, standardized mean difference; CI, confidence interval; I², inconsistency statistic.*
