# Supplementary material for: Oral-fluid inflammatory and oxidative-stress biomarkers for peri-implantitis in Asian adults: a systematic review and stratified random-effects meta-analysis
Source: Open Life Sci. 2026 Oct 16;21(1):20251360. doi: 10.1515/biol-2025-1360 (PMC13674786; doi:10.1515/biol-2025-1360)
Supplement: Supplementary file 2 [file j_biol-2025-1360_suppl_003.docx]

Figure S1: The results of the sensitivity analysis
